# The clinical safety, biodistribution and internal radiation dosimetry of [^18^F]AH113804 in healthy adult volunteers

**DOI:** 10.1186/s13550-016-0239-y

**Published:** 2016-11-29

**Authors:** E. J. Somer, R. Owenius, A. Wall, G. Antoni, A. Thibblin, J. Sörensen

**Affiliations:** 1 GE Healthcare, Life Sciences, Imaging R&D, Amersham, UK; 2 Department of Surgical Sciences, Radiology, Uppsala University, Uppsala, Sweden; 3 Department of Medicinal Chemistry, Preclinical PET Platform, Uppsala University, Uppsala, Sweden; 4 GE Healthcare, Life Sciences, Imaging R&D, Uppsala, Sweden

## Abstract

**Background:**

Quantitative biodistribution, venous blood and excretion data have been obtained following the intravenous bolus injection of AH113804 (^18^F) Injection in six healthy volunteers (HVs), four males and two females, up to approximately 5 h post-injection.

For each subject, key organs and tissues were delineated and analytical fits were made to the image data as functions of time to yield the normalised cumulated activities. These were input to an internal radiation dosimetry calculation based upon the Medical Internal Radiation Dose (MIRD) schema for the Cristy-Eckerman adult male or female phantom. The absorbed doses per unit administered activity to the 24 MIRD-specified target organs were evaluated for an assumed 3.5-h urinary bladder voiding interval using the Organ Level INternal Dose Assessment/Exponential Modelling (OLINDA/EXM) code. The sex-specific absorbed doses were then averaged, and the effective dose per unit administered activity was calculated.

**Results:**

Excluding the remaining tissue category, the three source regions with the highest mean initial ^18^F activity uptake were the liver (18.3%), lung (5.1%) and kidney (4.5%) and the highest mean normalised cumulated activities were the urinary bladder contents and voided urine (1.057 MBq h/MBq), liver (0.129 MBq h/MBq) and kidneys (0.065 MBq h/MBq). The three organs/tissues with the highest mean sex-averaged absorbed doses per unit administered activity were the urinary bladder wall (0.351 mGy/MBq), kidneys (0.052 mGy/MBq) and uterus (0.031 mGy/MBq).

**Conclusions:**

AH113804 (^18^F) Injection was safe and well tolerated. Although the effective dose, 0.0298 mSv/MBq, is slightly greater than for other common ^18^F PET imaging radiopharmaceuticals, the biodistribution and radiation dosimetry profile remain favourable for clinical PET imaging.

## Background

The tyrosine-kinase receptor c-Met (also known as MET) and its ligand, the hepatocyte growth factor (HGF), have been shown to be involved in tumour growth, invasion and metastasis in many human cancers of epithelial origin [1]. This makes c-Met a potential target for molecular imaging diagnostics and therapy.

A recent in vivo study to visualise c-Met expression was carried out using the engineered anticalin molecule PRS 110, with monovalent specificity for c-Met, radiolabelled with ^89^Zr to asses specific uptake in different human tumour xenograft models [2]. This study showed a dose-dependent specific tumour uptake of [^89^Zr]PRS-110 in the c-Met-expressing H441 (non-small cell lung cancer) and U87-MG (primary glioblastoma) tumours, whereas the uptake was lower (similar to non-specific control uptake) in the c-Met-negative A270 (ovarian cancer) tumour model.

**Fig. 1:**
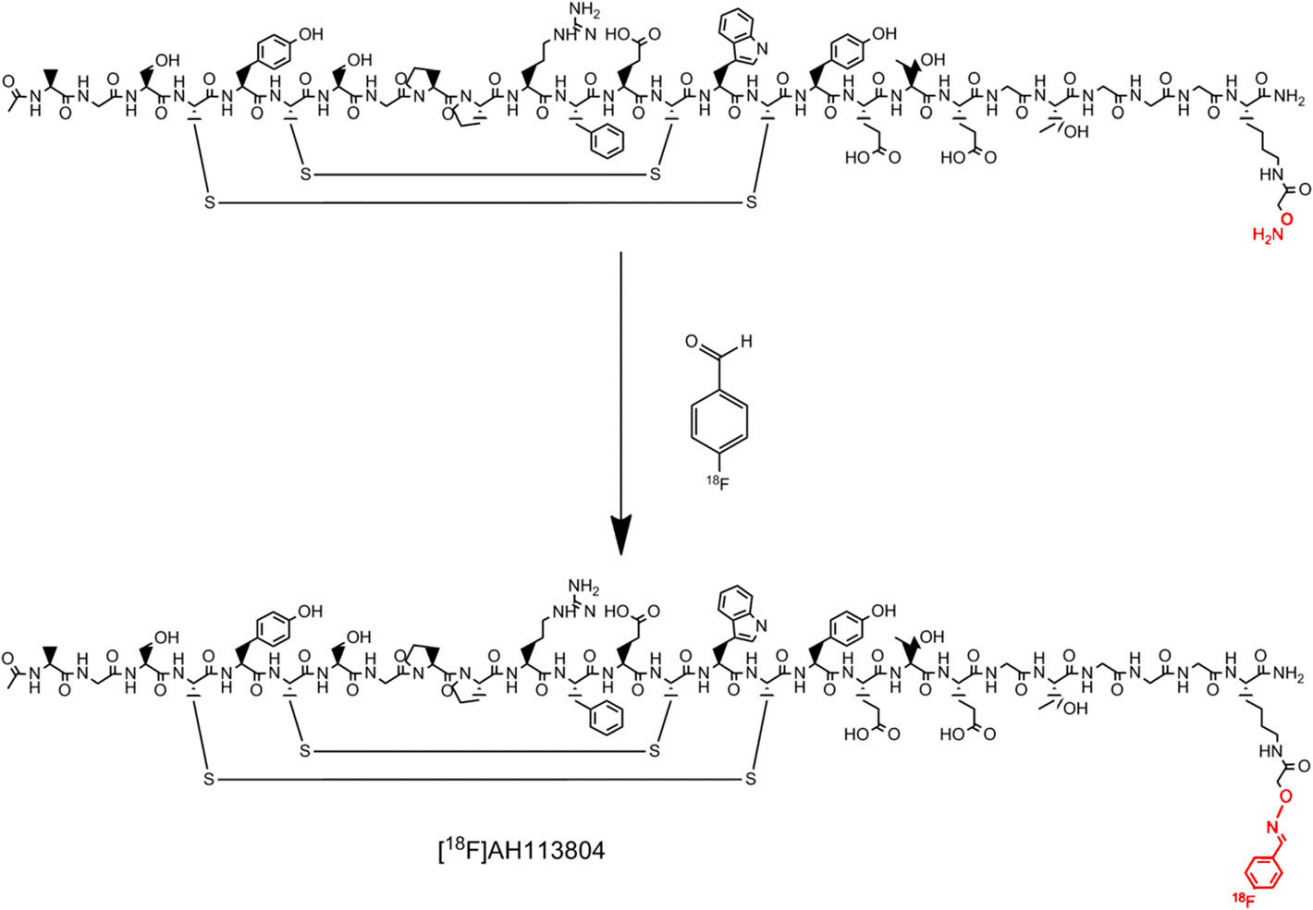
Synthesis scheme of [^18^F]AH113804

AH113804 (^18^F) Injection is an investigational, c-Met-targeted positron emission tomography (PET) imaging agent under development for the detection and quantification of c-Met expression. The [^18^F]AH113804 molecule consists of a 26-amino acid cyclic peptide (AH111972) that is conjugated with 4-[^18^F]fluorobenzaldehyde (Fig. 1). The synthesis of [^18^F]AH113804 has been described by Arulappu et al., together with an in vivo analysis of the utility of [^18^F]AH113804 for the detection of loco-regional recurrence of basal-like breast cancer in a mouse model [3].

A fluorescent cyanine-dye-labelled version of AH111972 (GE-137) has also been evaluated in a clinical phase 1 study in 20 healthy volunteers and 15 subjects with high risk of colorectal cancer [4]. In preparation of that study, GE-137 was shown to bind with high affinity to human c-Met with a dissociation constant, *K*_d_, of 3.0 ± 0.5 nM in a fluorescence polarisation binding assay and 2.7 ± 1.5 nM in a cell-based ligand binding assay with MKN45 cells that highly express human c-Met.

In addition, conjugation with 4-fluorobenzaldehyde (AH113804) was found to have no relevant effect on the binding of AH111972 to human c-Met (*K*_i_ of unlabelled AH113804 was not significantly different to that of AH111972 tested against GE-137 in a fluorescence polarisation competition assay; data not shown).

The intended use of AH113804 (^18^F) Injection is for PET imaging of c-Met expression in cancerous lesions with a potential objective to provide guidance in the selection of therapy and motivate the inclusion of c-Met inhibitors with the current standard therapy for additional benefit to the patient. PET imaging with the ^18^F-labelled glucose analogue fluorodeoxyglucose ([^18^F]FDG) was recently used to monitor the treatment response of crizotinib, a c-Met inhibitor designed to induce apoptosis, in mice with solid tumours [5]. However, in line with the conclusion of Newbold and co-workers, it would be advantageous to use a direct PET measure of apoptosis or c-Met expression in the monitoring of response to such drugs in addition to the metabolic assessment provided by [^18^F]FDG.

The objectives of this work were to assess the safety of a single dose of AH113804 (^18^F) Injection in healthy volunteers, to determine the biodistribution of ^18^F following intravenous (iv) administration of AH113804 (^18^F) Injection and to calculate the internal radiation dosimetry and the effective dose in healthy volunteers.

## Methods

Unless otherwise stated, all numerical data are provided as the mean ± 1 standard deviation.

### Radiopharmaceutical preparation

The target compound [^18^F]AH113804 was prepared in a two-step route starting with synthesis and purification of 4-[^18^F]fluorobenzaldehyde from 4-(trimethylammonium)benzaldehyde triflate, followed by the reaction of the labelled aldehyde with the deprotected peptide precursor (Fig. 1).

[^18^F]Fluoride anion was produced via the ^18^O(p,n)^18^F nuclear reaction using a Scanditronix MC17 cyclotron. The bombardment of enriched ^18^O water (98%, Rotem) gave an aqueous solution of ^18^F^−^ which was transferred from the cyclotron target using helium. The ^18^F^−^ (30–50 GBq) was trapped on a QMA column and eluted with kryptofix K222 and aqueous potassium hydrogen carbonate in acetonitrile (5.1 mg K222, 320 μL MeCN, 1.4 mg KHCO_3_, 80 μL water). This mixture was dried at 105–120 °C for 9.2 min. The aldehyde precursor (3.8 mg) dissolved in 2 mL of dry DMSO was added to the dried ^18^F^−^ kryptofix complex. The reaction mixture was heated at 80 °C for 2 min. 4-[^18^F]Fluorobenzaldehyde was purified with an Oasis MCX plus extraction cartridge using 4 mL of 4% aqueous ammonia and eluted in 1 mL of ethanol.

Five-milligramme peptide in 2.2 mL of aniline hydrochloride (10 mg/mL) was added to the purified labelled aldehyde. After 6.9 min at 60 °C, the crude product was diluted with sterile water and purified on a preparative HPLC column (Waters Xbridge Shield, 5 μm, 10 × 100 mm column with MeCN in 50 mM ammonium acetate, flow 4 mL/min; 0–1 min 15% MeCN, 1–16 min gradient 15–40% MeCN, 16–20 min gradient 40–100% MeCN) connected to a Knauer UV detector and a Bioscan RAD detector. Radiosynthesis was carried out on a FASTlab synthesiser module (GE Healthcare) using a single-use disposable AH113804 (^18^F) Injection FASTlab cassette. The product was formulated in 21 mL of phosphate-buffered saline containing sodium p-aminobenzoate (2.38 mg/mL). Sterile filtration was done on a Fluorodyne® 25-mm syringe filter (0.2 μm) (Pall Corporation, USA). The activity of the product was approximately 250 MBq/mL. The tracer production, including the drying of the [^18^F]fluoride anion and formulation of the purified product, took about 1 h.

The product identification and purity check was done by analytical reversed-phase HPLC utilizing a Phenomenex Kinetex C18, 2.6 μm, 100 × 4.6 mm column equipped with a Security Guard Ultra C18 cartridge with 10 mM ammonium acetate buffer (A) and methanol/acetonitrile 70:30 (*v*/*v*) (B). The gradient method was as follows: 0–3 min 30% B, 3–5 min 30–40% B, 5–32 min 40% B.

### Subjects

Six healthy volunteers (four men and two women) were enrolled. The age, whole body weight and body mass index (BMI) were 22 ± 2 years, 84 ± 10 kg and 27 ± 4 kg/m^2^, respectively (Table 1). The subject inclusion criteria included age greater than 18 years, the ability to provide informed written consent, normal medical history, physical examination and vital-sign findings, and a BMI between 18 and 30 kg/m^2^. The subject exclusion criteria included pregnancy and current lactation.

**Table 1:** Subject demographics and administered activity. Body mass index = weight[kg]/height[m]^2^

Subject	Sex	Age (year)	Weight (kg)	Height (cm)	Body mass index (kg/m^2^)	Administered activity (MBq)
1	Male	21	68.5	182	21	140
2	Female	21	91.2	177	29	143
3	Male	21	78.6	175	26	137
4	Male	26	79.9	184	24	135
5	Male	21	95.0	179	30	140
6	Female	23	92.0	174	30	154
Mean	–	22	84.2	178	27	142

### Administration

AH113804 (^18^F) Injection was administered intravenously as a slow (approximately 0.5–1 mL/s) bolus injection followed by a 10-mL saline flush. The mean administered activity was 141.5 ± 6.7 MBq with a mean injected volume of 1.8 ± 0.4 mL.

### Safety data

Safety data were collected up to 24 h after injection and included adverse events (AEs), vital signs (blood pressure, respiratory rate, heart rate and body temperature), physical examination (lungs, cardiovascular system and abdomen), electrocardiogram, laboratory parameters (serum biochemistry, haematology, coagulation parameters and urinalysis) and injection site status. Two-millilitre venous blood samples were collected through an indwelling catheter at a nominal 2-, 5-, 10-, 15-, 30-, 60-, 90-, 180- and 260-min post-injection, and ^18^F activity concentration in a single sample of whole blood and plasma was determined in a well counter (in-house design including 3MW3/3 detector, Saint-Gobain Crystals) that was cross-calibrated against the scanner and subject to daily quality control.

### Image acquisition and reconstruction

Emission images were acquired in 3D mode on a GE Discovery ST PET/CT scanner with a 15.7-cm axial field of view (FOV). A whole-body CT image was acquired for attenuation correction prior to the administration of AH113804 (^18^F) Injection. The axial extent of the acquired emission images was from the crown of the head to approximately mid-thigh so as to ensure that the urinary bladder was included in the image. Hence, a whole-body emission scan consisted of contiguous static positions acquired at a variable number of bed positions, depending on the subject’s height. Any ^18^F activity outside the FOV was assumed to be uniformly distributed throughout the unimaged anatomy.

Eight serial 3D whole-body PET images were acquired for each subject beginning nominally at 2 min and ending up to 6 h post-injection (p.i.). In order to compensate for the physical decay of ^18^F activity during imaging, the acquisition time for each bed position was increased from 30 to 60 s at 26 min and to 120 s at 220 min p.i. Images were acquired in three separate sessions between which the subjects were allowed to leave the scanner bed. Prior to the second and third emission imaging sessions, additional whole-body CT attenuation correction scans were performed. Corrections for scatter events and random coincidences were performed as per the models provided by the scanner’s manufacturer.

Subjects were encouraged to void and were given a yoghurt drink between imaging sessions to prompt gallbladder drainage into the duodenum. All urinary voids were collected and the volumes and ^18^F activity concentrations measured.

Emission images were reconstructed with both ordered subset expectation maximisation (OSEM) with 2 iterations and 21 subsets with post-reconstruction smoothing using an isotropic Gaussian filter (4.29 mm full-width at half-maximum) and filtered back-projection (FBP). Slice thickness was 3.27 mm with a pixel size of 3.91 mm in the OSEM reconstruction and 5.47 mm in the FBP.

### Quantification of activity

Image analysis was performed on a MIM workstation (version 6.0, MIM Software Inc., Cleveland) which includes a tool for volume of interest (VOI) definition using a predefined VOI template and an image registration algorithm. A set of VOIs were initially drawn around organs that could be readily delineated on the CT component of the PET/CT scan. These organs included the brain, salivary glands, thyroid, lungs, heart, liver, spleen and kidneys. The defined VOIs were registered to a common CT template using a non-rigid deformation algorithm and stored in the MIM database. This database was augmented as delineation was performed on successive subjects.

Analysis regions were defined using the templatized VOI database and were then manually edited using the fused CT and OSEM PET images as a guide. Having defined a VOI set for imaging session 1, these were translated as required and applied to sessions 2 and 3. Finally, VOIs were applied to the FBP data and the resulting mean activity concentration (Bq/mL) per region, the region volume (mL) and the standard deviation of counts within each region at each time point were exported in spreadsheet format. It was assumed that organs did not change in size or shape during the course of the acquisition with the notable exceptions of the bladder and the intestinal contents.

As the contents of the small intestine varied with time, VOIs were drawn on each time frame, and where no significant uptake could be identified, the VOI from the nearest available time point was copied to provide a measure of background activity. Background activity was included in the fitted model and subtracted from the resulting calculation of normalised cumulated activity and injected fraction entering the small intestine.

The activity within the cardiac chambers was estimated from the product of the measured whole blood activity and the chamber volume (477 mL for males, 351 mL for females representing 9% of the total blood volume) [6]. The activity within the cardiac wall was then estimated by subtracting the activity within the cardiac chambers from the activity within a whole heart VOI.

The presence of significant artefacts in the region of the bladder on both the FBP and OSEM reconstructed images complicated the analysis and bladder activity was estimated in two ways.

The first method used a 42% maximum intensity threshold VOI drawn on each time frame. The second method assumed the bladder was the only significant source within a rectangular VOI positioned to include the whole FOV over the maximum axial extent of the bladder. This whole-slice VOI was not resized as the bladder filled to ensure that the background component of the measured activity was as constant as possible. The change in background activity with time as a consequence of biological clearance was considered insignificant compared to the change in bladder activity per se. This background activity was then accounted for by including a constant term in the model used for curve fitting.

Administered activity not accounted for by the defined VOIs or excretion was assigned to the remainder category.

Measured activity data for each source region, *r*_s_, were decay-corrected to the time of injection and normalised to the administered activity. These data were then fitted to the generalised analytical function of Eq. 1,

1 $$ {A}_{r_{\mathrm{S}}}^{\mathrm{Corr},\mathrm{Norm}}(t)={C}_{r_{\mathrm{S}}}+{\displaystyle \sum_{i=1}^N{\alpha}_{r_{\mathrm{S}},i}\;{e}^{-{\lambda}_{r_{\mathrm{S}},i}t}} $$

where ${\alpha}_{r_{\mathrm{S}},i}$ and ${\lambda}_{r_{\mathrm{S}},i}$ are parameters extracted from a Simplex (GRG Nonlinear Solver, Microsoft Excel) fit minimising the weighted sum of squared difference between the model and the biodistribution data. The constant term, ${C}_{r_{\mathrm{S}}}$, was either fixed to zero or fitted alongside the other parameters. Mono- (*N* = 1) and bi-exponential (*N* = 2) fits were performed with and without the inclusion of the constant background term.

The selection of the appropriate equation to fit to the data was made using the Akaike Information Criterion (AIC) [7]. Fitted functions were subsequently integrated, after first accounting for the effect of ^18^F physical decay, to yield the normalised cumulative activities (NCAs) of the VOI [8]. The NCA of the urinary bladder contents and voided urine was calculated from the analytical fit to the summed activities in the urinary bladder contents and voided urine using a dynamic urinary bladder model [9]. As recommended by the International Commission on Radiological Protection (ICRP), a 3.5-h voiding interval was assumed [10].

### Internal radiation dosimetry

The internal radiation dosimetry for each subject was determined following the Medical Internal Radiation Dose (MIRD) schema [11]. For each subject, the NCAs were used as input to the Organ Level Internal Dose Assessment/Exponential Modelling (OLINDA/EXM) software [12] to calculate the absorbed doses to the 24 MIRD-specified target regions of the Cristy-Eckerman adult hermaphrodite male and adult female phantoms [13].

Following the recommendations of Publication 103 of the ICRP [14], these absorbed doses were then sex-averaged and the effective dose was evaluated using the tissue weighting factors of Publication 60 of the ICRP [15]. Recommendations of the ICRP subsequent to this publication were followed in the effective dose evaluation: the absorbed dose to the thymus gland was used as a surrogate for that to the oesophagus; the absorbed dose to the colon wall was calculated as the mass-weighted sum of the absorbed doses to the walls of the upper and lower large intestines; and the gonadal absorbed dose was taken to be the mean of the absorbed doses to the testes and ovaries [16].

## Results

Unless otherwise stated, all ^18^F activities are decay-corrected to the time of injection and expressed as a percentage of the administered activity.

### Safety

No AEs were reported and no clinically significant trends were noted for any safety parameter after administration as summarised below.

1. No deaths, serious AEs or clinically significant AEs were reported during the study. No study-emergent AEs were reported for any subject during the study.
2. No clinically significant changes in mean values of haematology, biochemistry, coagulation or urinalysis parameters were evident. In addition, no clinically significant shifts in individual laboratory values for healthy volunteers were noted.
3. No ECG abnormalities (changes) or trend indicative of an adverse safety signal were noted.
4. No clinically significant changes in vital signs parameters in healthy volunteers were noted.

### Biodistribution

**Fig. 2:**
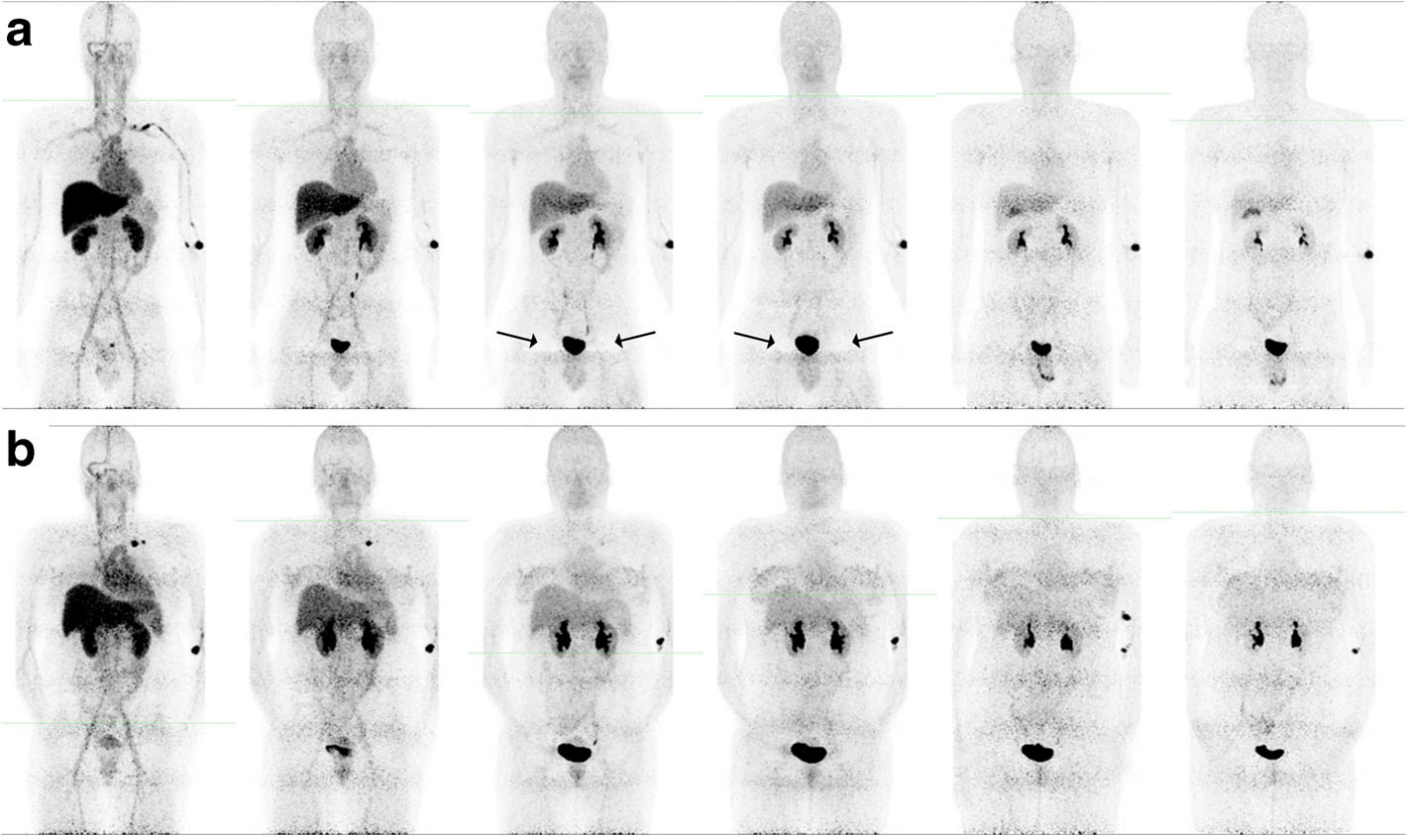
Representative PET Maximum Intensity Projections (MIP) following administration of AH113804 (^18^F) Injection. Representative OSEM PET Maximum Intensity Projection (MIP) of **a** subject 4 and **b** subject 2 following administration of AH113804 (^18^F) Injection. Acquisitions shown were performed at approximately (*left to right*) 0, 0.2, 0.5, 1.0, 2.5 and 3.7 h p.i. and features of note include the injection site on the left arm, the initial uptake in the heart and liver, clearance through the kidneys and the appearance of the gallbladder in later time frames. In subject 2, artefacts around the bladder are also apparent where the bladder activity is greatest

Figure 2 shows examples of PET images in male and female subjects over the course of the study following the administration of AH113804 (^18^F) Injection.

Excluding the remaining tissue category, the three source regions with the highest mean initial ^18^F activity uptake (defined as that at the initial imaging time point which was nominally 2 min p.i.) were the liver at 18.3 ± 2.9% (range 15.6–23.8%), the lung at 5.1 ± 0.8% (range 3.9–5.9%) and the kidney at 4.5 ± 0.5% (range 3.8–5.1%). Initial uptake in the brain, gallbladder, salivary glands, thyroid and cardiac wall was less than 1% of the injected activity in each of these organs/tissues.

**Fig. 3:**
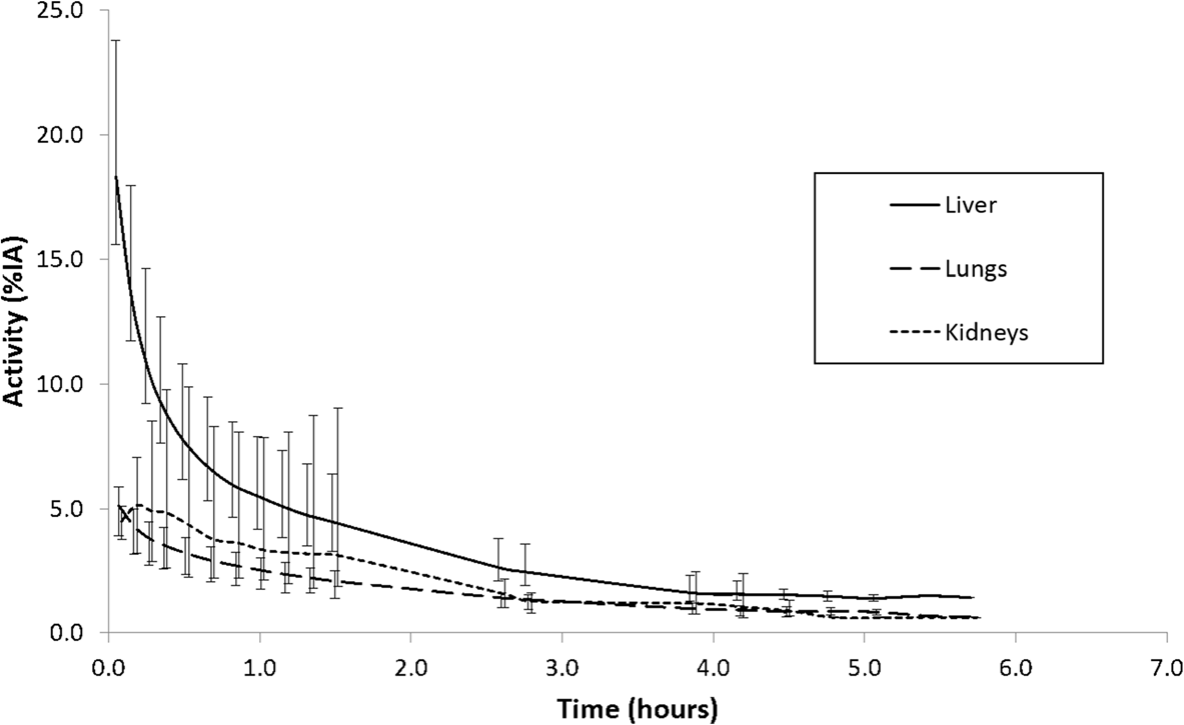
Time-activity curves for organs exhibiting the greatest uptake. Mean time-activity curves for the liver, lung and kidney being the organs with the highest initial uptake. Activity is corrected for physical decay and normalised to the injected dose. *Error bars* represent the range of the data and points have been offset slightly on the *x*-axis for clarity

Washout of ^18^F activity from the liver was generally rapid, falling to approximately 30% of its initial value at 1 h p.i. and less than to 10% of the initial value at 4 h p.i. Similarly, washout of ^18^F activity from the lungs was also rapid, reaching approximately 50% of its initial value at 1 h p.i. and 20% at 4 h p.i. The mean time-activity curves for the liver, lung and kidney for all subjects are shown in Fig. 3.

**Fig. 4:**
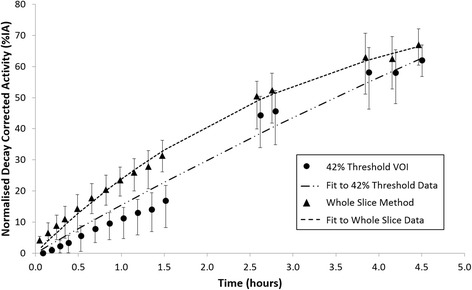
Time-activity curves for the urinary bladder illustrating the activity underestimation of the threshold VOI. Mean time-activity curves for the urinary bladder plus the measured voided activity determined from the 42% threshold VOI and the whole slice method. *Error bars* indicate the range of the data, and data has been offset slightly on the *x*-axis for clarity. The *broken lines* show the results of fitting the function *A*(*t*) = *C* + *α e* ^− *λt*^ (see Eq. 1). In the case of the whole slice region (*C* = 88, *α* = −88 and *λ* = 0.3131), the percentage of activity excreted through the renal pathway is 88%, and for the threshold VOI (*C* = 266, *α* = −266 and *λ* = 0.0598), the fitted parameters are not physiologically meaningful (i.e. renal excretion of 266%)

There was rapid clearance and excretion of ^18^F activity, primarily through the renal pathway, with about 60% excreted within 4 h after injection. Figure 4 illustrates the difference between the two methods used to evaluate the activity of the bladder contents plus voids. An attempt to model the data from the subject averaged data from the threshold VOI suggests an impossible total renal excretion of 266% of the injected activity. The whole-slice method yields more reasonable results, with total renal excretion of 88%.

**Fig. 5:**
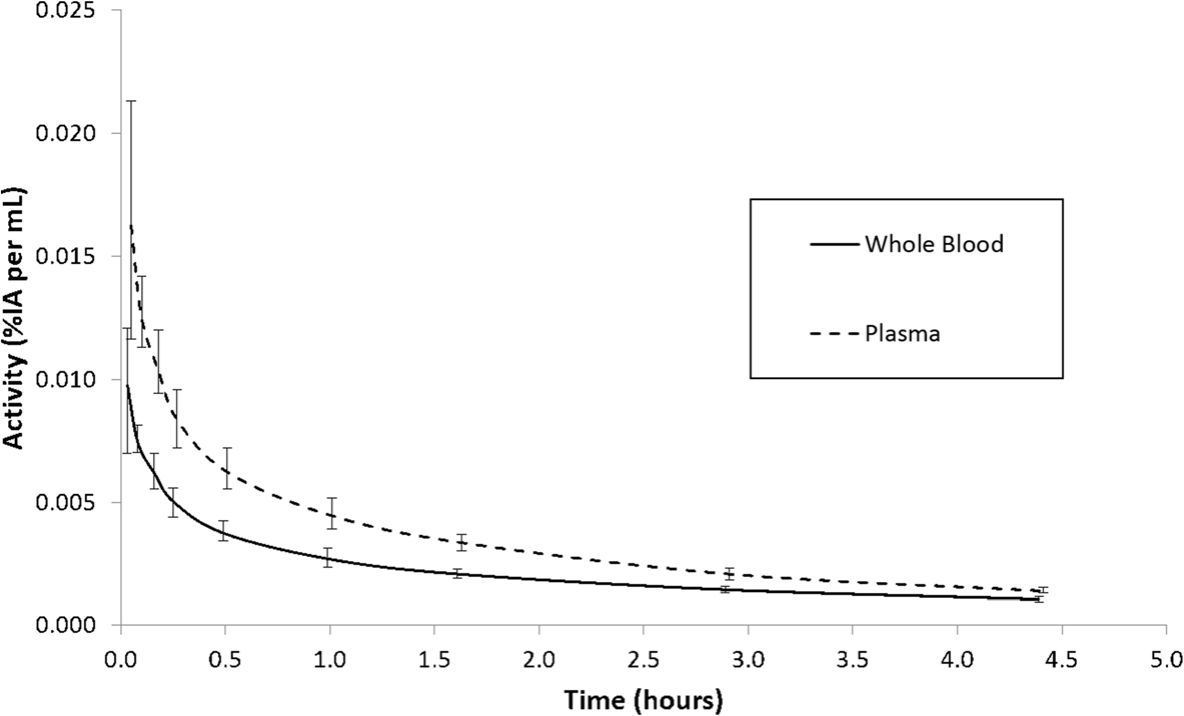
Normalised whole blood and plasma time-activity curves. Normalised whole blood and plasma time-activity curves for the five subjects where blood sampling was possible. The *error bars* indicate the range of the data and have been offset slightly for clarity

The ^18^F activity concentration in whole blood was characterised by bi-exponential decay with a 65% redistribution component (*T*_½_ = 6 min) as blood and tissue equilibrate and a 35% washout component (*T*_½_ = 2.1 h). In plasma the redistribution component was 63% (*T*_½_ = 6 min) and the washout component was 37% (*T*_½_ = 1.7 h). The slightly slower washout of activity from whole blood meant the ratio of whole blood activity to plasma activity rose from an initial value of 0.6 to 0.75 at 4.4 h p.i. The mean washout of activity from whole blood and plasma is shown in Fig. 5.

Blood sampling could not be carried out for subject 6 due to a malfunctioning sampling cannula, so instead, a weighted average of the other five subjects data was used as approximated blood and plasma curves [17]. The negative implications from this approximation were assessed as minor since data were only used in the estimation of the dose to the heart.

Table 2 summarises the NCA values determined in this study. If the remaining tissues category is excluded, the three source regions with the highest mean normalised cumulated activities were the urinary bladder (assuming 3.5 h void interval) with 0.589 ± 0.062 MBq h/MBq (range 0.500–0.652), the liver with 0.129 ± 0.024 MBq h/MBq (range 0.109–0.174) and the kidneys with 0.065 ± 0.032 MBq h/MBq (range 0.041–0.128).

**Table 2:** Normalised cumulative activity (NCA) for organs and tissues of significant uptake

Source region *r*_S_		$$ {\tilde{A}}_{r_{\mathrm{S}}}^{\mathrm{Norm}}\kern0.24em \left(\raisebox{1ex}{$ MBq h$}\!\left/ \!\raisebox{-1ex}{$ MBq$}\right.\right) $$							
		Subject						Mean	
		001–0011	001–0012	001–0013	001–0014	001–0015	001–0016		
Sex		M	F	M	M	M	F	M	F
Brain		1.02E−02	8.37E−03	1.02E−02	1.05E−02	8.29E−03	7.26E−03	9.78E−03	7.81E−03
Gallbladder		2.19E−03	5.74E−04	2.20E−03	2.84E−03	1.71E−03	5.53E−04	2.23E−03	5.64E−04
Heart contents		2.87E−02	2.27E−02	3.41E−02	3.10E−02	2.78E−02	2.53E−02	3.04E−02	2.40E−02
Heart wall		8.38E−03	1.67E−02	4.11E−03	6.37E−03	2.67E−03	1.37E−02	5.38E−03	1.52E−02
Kidneys		4.13E−02	1.28E−01	4.92E−02	5.04E−02	5.97E−02	6.24E−02	5.02E−02	9.52E−02
Liver		1.29E−01	1.27E−01	1.74E−01	1.24E−01	1.09E−01	1.11E−01	1.34E−01	1.19E−01
Lungs		5.99E−02	3.95E−02	5.05E−02	6.16E−02	5.62E−02	5.25E−02	5.71E−02	4.60E−02
Intestinal contents	Small intestine	2.20E−03	0.00E+00	6.35E−04	0.00E+00	3.73E−03	2.70E−03	1.64E−03	1.35E−03
	Bolus (unitless)	3.53E−03	0.00E+00	1.07E−03	0.00E+00	6.95E−03	5.38E−03	2.89E−03	2.69E−03
Spleen		1.78E−02	1.62E−02	1.66E−02	1.38E−02	2.00E−02	1.43E−02	1.71E−02	1.52E−02
Thyroid		6.61E−04	5.35E−04	9.35E−04	1.07E−03	6.17E−04	8.55E−04	8.20E−04	6.95E−04
Urinary bladder contents	Fit	1.14E+00	1.09E+00	8.87E−01	9.13E−01	1.14E+00	1.15E+00	1.02E+00	1.12E+00
	Dynamic model (3.5-h voiding interval)	5.94E−01	6.15E−01	5.00E−01	5.29E−01	6.41E−01	6.52E−01	5.66E−01	6.34E−01
Remaining tissues		1.20E+00	1.23E+00	1.32E+00	1.43E+00	1.25E+00	1.12E+00	1.30E+00	1.17E+00

The ‘Bolus’ value for the intestinal contents is the maximum decay-corrected fraction of injected activity encompassed by the intestinal VOI and is input to the ICRP 30 model within the OLINDA/EXM software

**Fig. 6:**
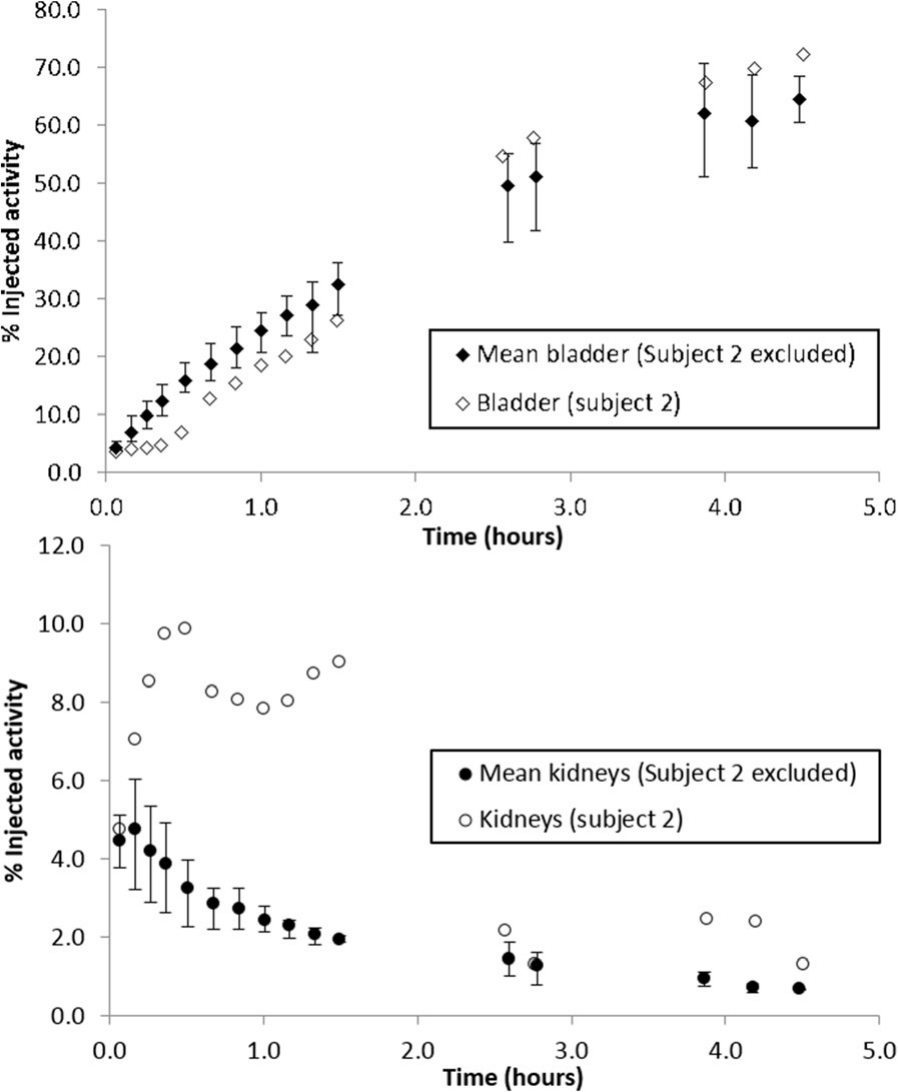
Time-activity curves to illustrate the delayed drainage of urine from the kidney to bladder in subject 2. Mean time-activity curves for the bladder and kidney for all subjects, except for subject 2, are presented with *error bars* to show the range of the data. Subject 2’s curves are also presented for comparison. The shape of the kidney curve in subject 2 is clearly abnormal and accounts for the high calculated NCA. The same subject’s bladder curve exhibits a corresponding reduction

The relatively high standard deviation in kidney NCA can be attributed to subject 2. In this female subject, tracer accumulated in the right kidney from approximately 60 min p.i. until the subject voided at the end of the first scan session (approximately 100 min p.i.). The bladder and kidney curves for this subject are presented in Fig. 6. This accounts for the high NCA in this subject’s kidney of 0.128 MBq h/MBq compared to the mean of the remaining subjects of 0.053 MBq h/MBq. As this represents a normal variant, no attempt was made to correct or exclude these data.

### Internal radiation dosimetry

The absorbed doses per unit administered activity to the MIRD-specified organs are provided in Table 3. The three organs/tissues with the highest mean sex-averaged absorbed doses per unit administered activity were the urinary bladder wall (0.351 ± 0.083 mGy/MBq), kidneys (0.052 ± 0.022 mGy/MBq) and uterus (0.031 ± 0.000 mGy/MBq).

**Table 3:** Male and female absorbed doses

Target region		$$ {\overline{D}}_{r_{\mathrm{T}}}^{\mathrm{Norm}}\kern0.24em \left(\raisebox{1ex}{$ mGy$}\!\left/ \!\raisebox{-1ex}{$ MBq$}\right.\right) $$					
		Male subjects			Female subjects		
		Mean	Minimum	Maximum	Mean	Minimum	Maximum
Adrenal glands		1.00E−02	9.39E−03	1.05E−02	1.28E−02	1.16E−02	1.40E−02
Brain		3.11E−03	2.80E−03	3.35E−03	3.25E−03	3.07E−03	3.42E−03
Breasts		6.05E−03	5.70E−03	6.52E−03	7.03E−03	6.76E−03	7.29E−03
Gallbladder wall		1.42E−02	1.29E−02	1.55E−02	1.33E−02	1.25E−02	1.41E−02
Gastrointestinal tract walls	Lower large intestine	1.55E−02	1.44E−02	1.66E−02	2.02E−02	2.02E−02	2.02E−02
	Small intestine	1.20E−02	1.11E−02	1.34E−02	1.41E−02	1.47E−02	1.44E−02
	Stomach wall	8.79E−03	8.26E−03	9.33E−03	1.10E−02	9.81E−03	1.04E−02
	Upper large intestine	1.13E−02	1.04E−02	1.26E−02	1.38E−02	1.43E−02	1.41E−02
Heart wall		1.45E−02	1.22E−02	1.56E−02	2.25E−02	2.16E−02	2.34E−02
Kidneys		3.58E−02	3.01E−02	4.14E−02	6.86E−02	4.68E−02	9.03E−02
Liver		2.02E−02	1.71E−02	2.51E−02	2.39E−02	2.22E−02	2.55E−02
Lungs		1.39E−02	1.31E−02	1.49E−02	1.51E−02	1.40E−02	1.62E−02
Muscle		8.74E−03	8.37E−03	9.16E−03	1.05E−02	1.01E−02	1.08E−02
Ovaries		1.50E−02	1.42E−02	1.58E−02	1.97E−02	1.95E−02	1.99E−02
Pancreas		1.03E−02	9.71E−03	1.08E−02	1.24E−02	1.15E−02	1.33E−02
Red marrow		7.98E−03	7.59E−03	8.42E−03	9.61E−03	9.21E−03	1.00E−02
Osteogenic cells		1.11E−02	1.04E−02	1.19E−02	1.34E−02	1.28E−02	1.40E−02
Skin		5.95E−03	5.62E−03	6.34E−03	6.90E−03	6.62E−03	7.17E−03
Spleen		2.24E−02	1.94E−02	2.55E−02	2.56E−02	2.36E−02	2.76E−02
Testes		1.15E−02	1.10E−02	1.19E−02	–	–	–
Thymus gland		7.63E−03	7.22E−03	8.23E−03	8.85E−03	8.55E−03	9.15E−03
Thyroid gland		8.78E−03	6.50E−03	1.27E−02	1.04E−02	8.66E−03	1.21E−02
Urinary bladder wall		2.74E−01	2.43E−01	3.09E−01	4.28E−01	4.16E−01	4.40E−01
Uterus		2.47E−02	2.28E−02	2.67E−02	3.08E−02	3.06E−02	3.09E−02
Total body		9.25E−03	8.87E−03	9.65E−03	1.13E−02	1.08E−02	1.17E−02

The sex-averaged effective dose per unit administered activity was evaluated and was determined to be 0.0298 mSv/MBq (Table 4).

**Table 4:** ICRP organ-weighting factors, sex-averaged absorbed and effective doses to ICRP 60 organs and total effective dose

ICRP 60 organ	Organ weight factor	Sex-averaged	
		Absorbed dose (mGy/MBq)	Effective dose (mSv/MBq)
Gonads	0.20	1.56E−02	2.88E−03
Bone marrow (red)	0.12	8.79E−03	1.06E−03
Colon	0.12	1.49E−02	1.79E−03
Lung	0.12	1.45E−02	1.74E−03
Stomach	0.12	9.60E−03	1.15E−03
Bladder	0.05	3.51E−01	1.76E−02
Breast	0.05	7.03E−03	3.27E−04
Liver	0.05	2.20E−02	1.10E−03
Oesophagus	0.05	8.24E−03	4.12E−04
Thyroid	0.05	9.58E−03	4.79E−04
Skin	0.01	6.42E−03	6.42E−05
Bone surface	0.01	1.22E−02	1.22E−04
Remainder: adrenals	0.05	1.14E−02	5.71E−04
Remainder: brain		3.18E−03	1.59E−04
Remainder: SI		1.32E−02	6.60E−04
Remainder: kidneys		5.22E−02	2.61E−03
Remainder: muscle		9.59E−03	4.80E−04
Remainder: pancreas		1.14E−02	5.68E−04
Remainder: spleen		2.40E−02	1.20E−03
Remainder: thymus		8.24E−03	4.12E−04
Remainder: uterus		3.08E−02	1.39E−03
Total effective dose	1.00		2.98E−02

## Discussion

This was a phase 1, single-centre, open-label study to evaluate the clinical safety, biodistribution of ^18^F and the internal radiation dosimetry associated with [^18^F]AH113804 in healthy adult volunteers. The radiopharmaceutical was found to be safe and well tolerated.

### Methodology

In this study the biodistribution was evaluated from PET/CT images of four male and two female subjects at up to 16 time points up to 4.5 h p.i. and the dosimetry evaluated using the OLINDA/EXM code.

High tracer concentration in the bladder, resulting from rapid renal excretion, caused significant artefacts in both FBP and OSEM reconstructed images. These took the characteristic form of radial streaks in FBP images or cold regions adjacent to the hot bladder in OSEM reconstructions. The assumptions that, despite these artefacts, the total number of counts recorded within each slice remains accurate and that changes in recorded activity are primarily due to the filling and emptying of the bladder while the background remains relatively constant, appear to hold true. These artefacts may be the result of a failure of the scatter correction algorithm in high count regions. Acquisition in 2D mode may have limited scatter thus reducing the appearance of these artefacts, but the consequent loss of scanner sensitivity may have led to longer acquisition times forcing data collection at fewer time points. Phantom experiments are required to confirm these assumptions.

### Biodistribution

The rapid clearance of tracer from blood and background tissue suggests that, having established the optimum imaging time p.i., dose optimisation may be possible while maintaining good image contrast. The change in the ratio of activity in whole blood to plasma suggests that immediately following injection, [^18^F]AH113804 is primarily distributed in plasma, but slowly accumulates in red blood cells as time progresses.

The mean NCA for the gallbladder in the female subjects was 75% less than in the males while kidney and bladder NCA were, respectively, 90 and 10% greater. The NCA to the heart wall was 183% greater for the females than the males. However, uptake in the gallbladder is relatively low and there is no difference in gallbladder absorbed dose between sexes. The difference in heart wall NCA may be attributed to errors in its estimation and the small sample size. The difference in NCA in the kidney and bladder does impact on the effective dose calculation as discussed below.

### Internal radiation dosimetry

The mean effective dose was more than 40% higher in female than male subjects, predominantly originating from the almost 60% higher absorbed dose to the urinary bladder wall and more than 90% higher absorbed dose to the kidneys in females. The high kidney absorbed dose to subject 2 (0.090 mGy/MBq) could be attributed to the retention of urine in the kidney as previously described but the kidney absorbed dose for subject 6 (0.047 mGy/MBq) is also greater than for any of the male subjects (mean 0.036 mGy/MBq) and the difference in effective dose between male and female subjects remains notable.

Gender-specific differences in effective dose occur in, for example, ^18^F-choline (0.027 mSv/MBq in males versus 0.037 mSv/MBq in females) and ^18^F-FLT (0.028 mSv/MBq in males versus 0.033 mSv/MBq in females) [18], but results are inevitably confounded by the small sample size. To place the calculated sex-averaged effective dose of 0.03 mSv/MBq in a clinical context, the effective dose for the most commonly used ^18^F-labelled PET tracer, FDG, is approximately 0.019 mSv/MBq [19].

## Conclusions

AH113804 (^18^F) Injection is a safe and well-tolerated PET radiopharmaceutical with a radiation dosimetry profile favourable for clinical PET imaging. Excretion of ^18^F was rapid and the highest absorbed doses were received by the urinary bladder wall, the kidneys and the uterus. The mean effective dose, 0.03 mSv/MBq, is slightly greater than for other common ^18^F PET imaging radiopharmaceuticals, but due to the dominant urinary excretion, future studies should ensure proper hydration of subjects to promote frequent voiding and minimise the absorbed radiation dose to the subject.
